# Pharmacists’ prescribing of opioids in Nova Scotia under Health Canada’s Controlled Drugs and Substances Act temporary exemption during the COVID-19 pandemic

**DOI:** 10.1177/17151635241267008

**Published:** 2024-08-14

**Authors:** Ronald Watema-Lord, Ingrid Sketris, Heather Neville, Mark Asbridge, Chiranjeev Sanyal

**Affiliations:** College of Pharmacy, Dalhousie University, Halifax, Nova Scotia; College of Pharmacy, Dalhousie University, Halifax, Nova Scotia; Drug Utilization Pharmacist & Pharmacy Research Coordinator, Nova Scotia Health, Halifax, Nova Scotia; Department of Community Health & Epidemiology, Dalhousie University, Halifax, Nova Scotia; College of Pharmacy, Dalhousie University, Halifax, Nova Scotia

## Introduction

The opioid epidemic is a public health crisis in the United States and Canada, with a surge in opioid prescriptions as a key contributor.^1,2^ During the coronavirus disease of 2019 (COVID-19) pandemic, the opioid crisis was exacerbated in Canada, with apparent opioid-related deaths increasing by 127% (8444 vs 4724) from 2020 compared to 2019.^3^ Pharmacists have addressed the opioid crisis through activities such as providing education, advancing harm reduction strategies and monitoring opioid use disorder (OUD).^3,4^ During COVID-19, public health directives to stay home, maintain social distancing and/or self-isolate created unique challenges for patients to access opioid therapy.^5^ The pandemic especially impacted marginalized communities, the elderly and those with OUD, among others.^3,6,7^

In addition, there were challenges in ensuring continuity of care for patients who were receiving opioids and other controlled drugs.^8^ Many Nova Scotia residents did not have access to a primary care physician and were self-isolating at home, many physicians had visits only virtually, emergency departments were closed or had limited hours because of staffing shortages and many elective surgeries were postponed.^9,10^ Recognizing this gap, on March 19, 2020, Health Canada issued a temporary exemption to the Controlled Drugs and Substances Act (CDSA) subsection 56(1) class.^11^ The purpose of this policy exemption was to maintain uninterrupted access to controlled substances, including opioids for medical treatment such as chronic pain and OUD, while Canadians were adhering to public health guidance.^11^ This exemption was extended for 5 years to September 30, 2026.^12^

The CDSA exemption allowed direct patient care pharmacists^13,14^ in Nova Scotia to prescribe, renew, extend and transfer prescriptions; deliver controlled medication prescriptions to patients; and accept verbal orders for controlled medication prescriptions within certain restrictions.^13,15^ Direct patient care pharmacists are those who have certified that they have practised sufficient direct patient care in pharmacy in the 2 preceding years to maintain competency in Nova Scotia.^14^ The Nova Scotia College of Pharmacists (NSCP) established an emergency protocol for prescribing narcotics and included additional provisions for safe prescribing of opioids by pharmacists for acute pain, opioid use disorder and other indications, which was later formalized into the standards of practice for prescribing drugs.^16^ To our knowledge, no studies have been conducted to assess the consequences of the CDSA exemptions on the new prescribing authority of pharmacists in Nova Scotia. The objective of this study was to describe opioids prescribed by direct patient care pharmacists in Nova Scotia to individuals 18 years or older following the exemption of CDSA subsection 56(1) during COVID-19.

## Methods

### Study design

This descriptive study used aggregate data generated by the Nova Scotia Prescription Monitoring Program (NSPMP) and provided to researchers.^17^ Aggregate data on opioids prescribed by direct patient care pharmacists (i.e., refills of existing prescriptions) to individuals 18 years or older at community pharmacies during COVID-19 in Nova Scotia were included. The study duration represents 2 fiscal years of the CDSA exemption period: April 1, 2020, to March 31, 2021 (period 1) and April 1, 2021, to March 31, 2022 (period 2). These 2 fiscal years were selected to describe the pattern of opioid prescribing by direct patient care pharmacists immediately after the enactment of the temporary exemption. Opioids were identified using the Anatomic Therapeutic Chemical (ATC) codes used by NSPMP. Opioids were classified into those used for analgesia and OUD based on common prescribing patterns and formulations. Opioid analgesics included in the study were codeine, fentanyl, hydromorphone, morphine, oxycodone, diphenoxylate, butorphanol, pentazocine, tapentadol, meperidine and tramadol. Buprenorphine and methadone to treat OUD were also included.

### Data source

The NSPMP monitors and maintains records of controlled drugs listed under the Controlled Drugs and Substances Act, including opioids.^18,19^ The NSPMP’s legislated mandate is “to promote the appropriate use of monitored drugs in Nova Scotia and to reduce the abuse or misuse of monitored drugs in the province.”^19^ The NSPMP captures information on all controlled drugs dispensed to individuals at community pharmacies across Nova Scotia and excludes opioid prescribing to hospital patients.^20^ All prescribers who prescribe monitored drugs, and all pharmacists and pharmacies that dispense monitored drugs, are required to register with the NSPMP.^21^ Prescriptions for monitored drugs are either written on specific NSPMP prescription pads or e-prescribed directly into a centralized Drug Information System.

### Data analysis

The data represent opioids prescribed by direct patient care pharmacists in Nova Scotia to individuals 18 years or older. NSPMP provided aggregate data to researchers with no possibility of identifying individuals. Sample sizes less than 5 were suppressed and collapsed into larger categories. The location of a community pharmacy was based on the first 2 letters of the postal code, with B0 codes identifying rural areas and B1–B9 codes identifying urban areas based on Canada Post’s forward sortation area.^22^ In addition, mean days supplied/prescription and mean prescriptions/patient were variables of interest and stratified by age group and sex. Continuous variables were analyzed using descriptive statistics and reported as means with standard deviations. Categorical variables were analyzed as frequencies and percentages. The dispensing claims data where the prescriber was identified by NSPMP as a direct patient care pharmacist were analyzed, hereafter referred to as dispensed. Data analysis was conducted using PL/SQL Developer (version 15.0.4.2064) and Microsoft Excel.

### Ethics approval

Ethics approval for the study was granted by the Dalhousie Health Sciences Research Ethics Board in compliance with the principles of the Tri-Council policies on Ethical Conduct for Research Involving Humans (#2023-6558).

## Results

The total number of direct patient care pharmacists who prescribed opioids out of all eligible pharmacists was 49.5% (577/1165) and 55.4% (663/1196) in period 1 and period 2, respectively. In period 1, 577 pharmacists dispensed a total of 3133 prescriptions, which increased to 5151 and 663 in period 2. The mean number of prescriptions dispensed per year by pharmacists experienced the greatest increase in rural areas and subsequent dispensations for analgesia in period 2 (76.3% and 67.2%, respectively) (Table 1).

**Table 1:** Opioid dispensations by pharmacists in Nova Scotia to individuals 18 years or older during the COVID-19 pandemic

Opioid dispensations by pharmacists	April 1, 2020, to March 31, 2021		April 1, 2021, to March 31, 2022		April 1, 2020, to March 31, 2021 vs April 1, 2021, to March 31, 2022	
	Total PPY	Mean (SD) PPY	Total PPY	Mean (SD) PPY	% Change (total PPY)	% Change (mean PPY)
Opioids used as analgesics (first dispensations)	1846	3.5 (4.0)	2619	4.5 (8.5)	41.9	26.1
Opioids used as analgesics (subsequent dispensations)	367	4.3 (7.3)	857	7.1 (20.4)	133.5	67.2
Opioids used as analgesics (first dispensations and subsequent dispensations combined)	2213	4.2 (5.8)	3476	5.9 (15.7)	57.1	33.1
Opioids used to treat OUD	920	4.6 (7.3)	1675	5.4 (7.9)	82.1	19.1
All opioids by geographic location of pharmacy (urban)*	2458	5.6 (8.7)	3717	7.1 (14.0)	51.2	27.3
All opioids by geographic location of pharmacy (rural)*	675	4.5 (5.1)	1434	7.9 (20.3)	112.4	76.3
All opioids by patient residence (NS cardholders)	3040	5.3 (8.0)	4934	7.6 (16.4)	62.3	42
All opioids by patient residence (non-NS cardholders)	93	1.9 (1.8)	217	2.6 (2.4)	133.3	37.4

NS, Nova Scotia; OUD, opioid use disorder; PPY, prescriptions per year; SD, standard deviation.

First dispensations include dispenses that are initial fills against refill renewal orders, a first fill where the quantity supplied is less than 1 full repetition of the ordered amount or emergency supply. Subsequent dispensations include dispenses that are a fill against an order that has already been filled (or partially filled) at least once, a refill where the quantity supplied is less than 1 full repetition of the ordered amount, or a fill providing sufficient supply for 1 day.

* B0 codes designated as rural areas and B1–B9 areas designated as urban areas based on Canada Post’s forward sortation area.

Hydromorphone accounted for the largest number of total opioids prescribed by pharmacists for analgesia over the study period: 22.7% (712/3133) and 25.7% (1325/5151) for period 1 and period 2, respectively. This was followed by oxycodone, 16.3% (509/3133) and 16.8% (863/5151), and morphine, 12.3% (386/3133) and 10.4% (536/5151), for period 1 and period 2, respectively. The opioid dispensed varied by opioid category across age groups. Hydromorphone and oxycodone were dispensed mostly to patients aged 50 to 64 years in period 1 compared to period 2 (49.6% [353/712] and 46.6% [618/1325]; 47% [239/509] and 45.4% [392/863], respectively). Methadone was dispensed mostly to patients aged 30 to 39 years, 34.5% (213/618) in period 1 and 37.3% (381/1022) in period 2.

The opioid prescriptions varied by opioid category across females and males for period 1 and period 2 (Table 2). Of note, fentanyl was prescribed more frequently to females: 58.1% (25/43) in period 1 and 61.2% (52/85) in period 2 (Table 2). The mean days supply to individuals 18 years or older by age group ranged from 1.0 to 29.7 in period 1 and 1.0 to 28.7 in period 2. For females and males, the mean days supply ranged from 2.6 to 22.3 and 2.2 to 23.9, respectively (Table 2).

**Table 2:** Opioid dispensations by patient sex, average duration and number of prescriptions in individuals 18 years or older in Nova Scotia during the COVID-19 pandemic

Opioid drug type	Patient sex	April 1, 2020, to March 31, 2021			April 1, 2021, to March 31, 2022		
		Number (%) of opioid prescriptions dispensed	Mean (SD) days supplied/Rx	Mean (SD) Rx/patient	Number (%) of opioid prescriptions dispensed	Mean (SD) days supplied/Rx	Mean (SD) Rx/patient
Buprenorphine	Females	115 (29.8)	6.1 (9.4)	1.9 (1.6)	246 (33.1)	3.4 (6.0)	2.7 (3.0)
	Males	271 (70.2)	3.6 (5.9)	2.7 (2.9)	497 (66.9)	2.6 (4.4)	3.4 (4.6)
Codeine	Females	247 (58.7)	21.4 (13.6)	1.4 (1.5)	284 (53.9)	20.8 (11.3)	1.4 (1.1)
	Males	174 (41.3)	21.8 (14.1)	1.5 (1.1)	243 (46.1)	22.6 (10.3)	1.4 (1.0)
Fentanyl	Females	25 (58.1)	18.8 (11.1)	1.3 (0.7)	52 (61.2)	23.9 (7.7)	2.7 (5.1)
	Males	18 (41.9)	20.6 (9.2)	1.1 (0.2)	33 (38.8)	21.5 (10.4)	1.8 (1.4)
Hydromorphone	Females	352 (49.4)	15.4 (11.3)	1.8 (2.0)	585 (44.2)	14.5 (11.4)	2.3 (4.7)
	Males	360 (50.6)	15.0 (11.7)	1.8 (1.7)	739 (55.8)	10.9 (12.0)	2.8 (10.5)
Methadone	Females	193 (31.2)	3.2 (5.0)	2.2 (2.8)	412 (40.3)	2.2 (3.6)	2.9 (5.3)
	Males	425 (68.8)	2.6 (4.1)	2.6 (4.4)	610 (59.7)	2.7 (3.9)	2.5 (4.1)
Morphine	Females	165 (42.8)	12.6 (12.1)	2.2 (2.6)	273 (50.9)	10.3 (13.1)	2.6 (4.7)
	Males	221 (57.3)	8.3 (10.9)	3.9 (6.8)	263 (49.1)	10.2 (11.2)	2.3 (4.4)
Oxycodone	Females	241 (47.4)	20.2 (12.4)	1.5 (1.1)	380 (44.0)	18.5 (11.7)	2.0 (2.6)
	Males	268 (52.7)	19.4 (11.2)	1.6 (1.8)	483 (56.0)	20.2 (11.3)	2.0 (2.6)
Other opioids	Females	38 (65.5)	22.3 (10.2)	1.5 (0.9)	35 (70.0)	17.6 (10.4)	1.5 (1.1)
	Males	20 (34.5)	16.6 (10.6)	1.7 (1.4)	15 (30.0)	14.7 (9.1)	1.1 (0.4)

Rx, prescription; SD, standard deviation.

Opioids were grouped into the “other opioids” category when numbers were too small to report. This category includes diphenoxylate, butorphanol, pentazocine, tapentadol, meperidine and tramadol.

## Discussion

The CDSA subsection 56(1) exemption enabled direct patient care pharmacists to prescribe opioids in Nova Scotia, Canada. Direct patient care pharmacists prescribed over 3000 opioid prescriptions from April 1, 2020, to March 31, 2021, following the CDSA subsection 56(1) exemption, which increased to over 5000 prescriptions from April 1, 2021, to March 31, 2022. The greatest increases were noted for subsequent dispensations and in rural areas.

During the COVID-19 pandemic, community pharmacies were considered an essential service and were open in Canada.^3,15^ Pharmacists were the most accessible health care provider and an important point of contact in the communities.^15^ Our study demonstrated that the temporary exemption enabled direct patient care pharmacists to prescribe opioids such that patients were able to access their therapy.

The most common opioids prescribed by pharmacists for analgesia were hydromorphone, followed by oxycodone and morphine. This was similar to other Canadian studies, where hydromorphone and oxycodone were the most prescribed opioids.^23,24^

The mean number of days supplied across age groups ranged from 1.0 (standard deviation [SD] 0.0) to 29.7 (SD 0.7). During COVID-19, there was a steep increase in demand for medications, and to minimize the risk of drug shortages, the Nova Scotia College of Pharmacists directed that a maximum 30-day supply should be provided.^15^

A study strength is the use of prescription claims data from the NSPMP database. Previous research has demonstrated prescription claims data have very high validity.^25^ The NSPMP is a population-based database that routinely collects comprehensive and reliable information on all monitored drugs dispensed at community pharmacies, including urban and rural locations in Nova Scotia. The data allowed us to examine first dispensation and subsequent dispensation by pharmacists.

There are various study limitations. To preserve patient confidentiality, aggregate data were provided, which only allowed descriptive analyses, and sample sizes less than 5 were collapsed into larger groups. Patient demographics, outcomes data, indication for use, and whether opioid prescribing was safe and appropriate are unknown and also limitations. In addition, the generalizability of the study findings to other contexts is unknown. Opioid prescription data may not reflect patterns of opioid dispensing or consumption. Due to the observational nature of the study, the effect of external factors on patterns of opioid prescribing is unknown.

Future research should examine whether the opioids prescribed by direct patient care pharmacists were safe and appropriate, as well as patient outcomes, patient satisfaction and naloxone prescribing by pharmacists. Pharmacists’ awareness of opioid prescribing guidelines, the extent of the patient counselling provided on risks and benefits of opioid therapy and monitoring of opioid therapy should also be examined. As well, the consequences of similar prescribing legislation exemptions to facilitate access to opioids or other restricted medications in other provinces of Canada or internationally would be of interest.

## Conclusions

This study indicated that Nova Scotian direct patient care pharmacists used the temporary exemption of CDSA subsection 56(1) during the COVID-19 pandemic to prescribe opioid therapy according to defined standards. This helped individuals in Nova Scotia access their opioid therapy, particularly those living in rural communities and those receiving buprenorphine and methadone. The types of opioids prescribed by pharmacists in Nova Scotia during the pandemic were documented, with hydromorphone being prescribed most frequently. Given the gaps in accessing primary care, these results should facilitate discussions on the vital role of pharmacists to support continuity of care for patients receiving opioid therapy. ■
